# Gamma oscillations predict pro-cognitive and clinical response to auditory-based cognitive training in schizophrenia

**DOI:** 10.1038/s41398-020-01089-6

**Published:** 2020-11-23

**Authors:** Juan L. Molina, Michael L. Thomas, Yash B. Joshi, William C. Hochberger, Daisuke Koshiyama, John A. Nungaray, Lauren Cardoso, Joyce Sprock, David L. Braff, Neal R. Swerdlow, Gregory A. Light

**Affiliations:** 1 Department of Psychiatry, University of California, San Diego, San Diego, CA USA; 2 Department of Psychology, Colorado State University, Fort Collins, CO USA; 3 VA Desert Pacific Mental Illness Research, Education and Clinical Center (MIRECC), VA San Diego Healthcare System, San Diego, CA USA

**Keywords:** Predictive markers, Schizophrenia

## Abstract

Cognitive impairments are pervasive and disabling features of schizophrenia. Targeted cognitive training (TCT) is a “bottom-up” cognitive remediation intervention with efficacy for neurocognitive outcomes in schizophrenia, yet individual responses are variable. Gamma oscillatory measures are leading candidate biomarkers in the development of biologically informed pro-cognitive therapeutics. Forty-two schizophrenia patients were recruited from a long-term residential treatment facility. Participants were randomized to receive either 1 h of cognitive training (TCT, *n* = 21) or computer games (TAU, *n* = 21). All participants received standard-of-care treatment; the TCT group additionally completed 30 h of cognitive training. The auditory steady-state response paradigm was used to elicit gamma oscillatory power and synchrony during electroencephalogram recordings. Detailed clinical and cognitive assessments were collected at baseline and after completion of the study. Baseline gamma power predicted cognitive gains after a full course of TCT (MCCB, *R*^2^ = 0.31). A change in gamma power after 1-h TCT exposure predicted improvement in both positive (SAPS, *R*^2^ = 0.40) and negative (SANS, *R*^2^ = 0.30) symptoms. These relationships were not observed in the TAU group (MCCB, SAPS, and SANS, all *R*^2^ < 0.06). The results indicate that the capacity to support gamma oscillations, as well as the plasticity of the underlying ASSR circuitry after acute exposure to 1 h of TCT, reflect neural mechanisms underlying the efficacy of TCT, and may be used to predict individualized treatment outcomes. These findings suggest that gamma oscillatory biomarkers applied within the context of experimental medicine designs can be used to personalize individual treatment options for pro-cognitive interventions in patients with schizophrenia.

## Introduction

Disruptions in neural oscillatory dynamics are thought to underlie the perceptual and cognitive impairment associated with schizophrenia (SZ)^1,2^. Gamma oscillations (30–80 Hz) have received much interest in preclinical and translational studies of SZ, given their role in local and interregional information flow critical for cognition and perception^3,4^. Gamma-band abnormalities in SZ are thought to arise from a disturbance in the activity of fast-spiking, parvalbumin-positive interneurons, and the consequent effects on the dynamic balance of excitation and inhibition in cortical microcircuits^5^. The resulting “mesoscale” abnormalities in gamma-band oscillations and synchrony have been proposed as pathophysiologic mechanisms underlying the cognitive and clinical symptoms of SZ^1,6^. Clinical studies in SZ patients have corroborated preclinical findings and have linked gamma-band abnormalities with disturbances in low-level sensory and perceptual processes^7–10^, psychopathological domains (e.g., hallucinations, disorganization, and thought disorder)^9–12^, and higher-order neurocognitive functions (e.g., working memory, executive function, verbal learning, and memory)^13–15^.

While evidence of aberrant gamma oscillations and synchrony in SZ has been documented extensively across a broad range of experimental paradigms, including task-based and resting-state conditions^12,15–18^, the heterogeneity of gamma-band abnormalities seen across experimental paradigms has limited the translation of these findings into clinical settings. From this perspective, the 40-Hz auditory steady-state response (ASSR) paradigm has emerged as a leading candidate biomarker in clinical and translational neuroscience, given its ability to provide robust measures of the brain’s capacity to support gamma oscillations under-optimized, stimulus-driven conditions^19–21^. In the ASSR paradigm, stimulus trains of amplitude-modulated tones or clicks presented at a rate of 40-Hz-drive oscillatory power and synchrony across a distributed thalamocortical network^22–25^. ASSR deficits in gamma oscillatory power and synchrony are well characterized in SZ^16,17,19^ and have been linked to pathophysiologic and phenomenological dimensions of psychotic disorders^12,22,26–30^ and other neuropsychiatric conditions.

Auditory-based targeted cognitive training (TCT) is a “bottom-up” cognitive remediation intervention designed to improve cognitive function by stimulating low-level perceptual networks presumed to mediate higher-order cognition, e.g., verbal memory and executive function. TCT has demonstrated efficacy in SZ and psychosis-spectrum disorders^31–34^ with enduring benefits in cognitive and functional outcomes^32,35^. Recent meta-analysis confirms the efficacy of TCT and other related cognitive remediation interventions on cognitive, clinical, and functional outcomes^36–38^. Despite the abundance of evidence demonstrating the feasibility, tolerability, and efficacy of TCT and other cognitive remediation interventions in psychotic disorders, the adoption of TCT in community settings remains stagnant (cf. Thomas et al.^33^). The variability of patient responses to TCT remains a significant barrier to the broader implementation of TCT and other pro-cognitive interventions in broader community settings. While TCT is efficacious at the group level, individual responses to TCT are variable with only subsets of patients showing clinically meaningful cognitive gains.

Electroencephalographic (EEG) biomarkers of early auditory information processing (EAIP) are promising tools in experimental medicine, which may help parse the heterogeneity of responses to TCT and other pro-cognitive interventions^39,40^. We have demonstrated the utility of EAIP biomarkers, including ASSR, as sensitive measures of target engagement and therapeutic sensitivity in neural mechanisms relevant for learning, memory, and cognitive rehabilitation^40–43^. Findings from these experimental medicine studies suggest that assessments of baseline (e.g., event-related activity recorded prior to the initiation of TCT or other pro-cognitive interventions) and the capacity for change or “malleability” of the underlying neural circuitry in response to acute or limited “doses” of TCT may predict future TCT-related outcomes^44,45^. We recently completed a “proof-of-concept” experimental medicine trial assessing the utility of EAIP biomarkers as predictors of cognitive and clinical response to TCT^33,46^. In Thomas et al.^33^, we report the main clinical outcomes from this trial, including improvement in verbal learning and reduction in positive symptoms in the TCT group relative to the treatment as the usual group. Consistent with the predictions of our experimental medicine framework, we found that both baseline and the magnitude of change in our primary EAIP biomarkers after 1 h of TCT or “EAIP malleability,” predicted improvement in cognition after a full (30-h) course of TCT in this cohort of chronic SZ patients^46,47^.

Despite the widespread use of ASSR in the clinical and translational literature, its use as a predictive biomarker of pro-cognitive response to TCT or other cognitive remediation interventions has not been evaluated. Here we present findings from our analysis of ASSR measures in a cohort of patients with chronic psychotic disorders previously characterized as part of our “proof-of-concept” experimental medicine study assessing the utility of EAIP biomarkers as predictors of TCT-related cognitive and clinical outcomes^33,46,47^. Given the capacity of ASSR measures to detect plasticity in cortical mechanisms of interest to cognitive remediation, we hypothesized that EEG measures of gamma oscillatory power and synchrony collected at the outset of treatment (e.g., “baseline” and “malleability” after 1 h of TCT) would predict pro-cognitive and clinical responses to TCT in refractory patients with chronic psychotic disorders.

## Materials and methods

### Participants and study design

Forty-two patients with treatment-refractory SZ or schizoaffective disorder were recruited from a community-based inpatient-treatment program following an extended acute-care hospitalization. Sample-size calculations were made to ensure adequate power to detect effect sizes (*d* ≈ 0.8) previously reported in Fisher et al.^31^ for primary outcomes in this trial. Details of the recruitment and ascertainment procedures in this cohort were previously reported^33,46^. Participants were enrolled in the study after they were determined to be clinically stable by the treatment team. All participants were under public conservatorship by the San Diego or Los Angeles Counties. The diagnosis was verified using an abbreviated version of the Structured Clinical Interview for DSM-IV-TR^48^. Exclusion criteria included premorbid intellectual disability (e.g., wide range achievement test (WRAT) reading subtest below 70), inability to provide informed consent, limited English proficiency, history of significant neurological illness or head injury, severe systemic illness, or current mania. All participants were engaged in rehabilitative programming as part of their standard of care or “treatment as usual” (TAU), including medication management, individual and group therapy, and participation in structured social activities. After initial screening and assessments, enrolled participants were randomized to receive either 1 h of cognitive training (TCT, *n* = 21) or 1 h of computer games (TAU, *n* = 21) using a parallel design with stratified random assignment by sex, age, and ethnicity. Participants randomized to TAU continued to receive standard-of-care treatment. The TCT group additionally completed 1 h of TCT, ~3–5 days per week, for 30 h. Neurophysiologic recordings were obtained at baseline (T0) and after participants underwent 1 h (T1) of TCT or 1 h of computer games (TAU). Structured clinical and cognitive assessments were collected at baseline (T0) and at the end of the study (T2, approximately 10–12 weeks later). All subjects provided written informed consent. The Institutional Review Board of the University of California, San Diego, approved all experimental procedures (IRB#130874).

### Targeted cognitive training

TCT was administered on individual laptop computers with headphones. We previously reported full details of the cognitive training exercises applied in TCT^33,46^. Briefly, six training exercises by BrainHQ (Posit Science Corporation, San Francisco, CA) were administered. Training exercises were designed to engage neuroplasticity mechanisms in auditory networks of auditory perception and processing speed (Sound Sweeps, Fine Tuning) and auditory memory (Syllable Stacks, Memory Grid, To-Do List Training, and Rhythm Recall). Exercises applied an n-up/m-down algorithm to participant responses to estimate thresholds, ensuring that participants were engaging in the exercises and were continuously challenged at an appropriate level (~80% accuracy) as their abilities improved.

### Clinical and cognitive assessments

Clinical and cognitive outcomes in the TCT vs. TAU groups from this cohort were previously reported^33^. Participants were assessed on measures of cognition and clinical symptoms at baseline (T0) and at the end of treatment (T2). Cognition was assessed using age- and gender-corrected T scores from the MATRICS consensus cognitive battery (MCCB)^49^. MCCB measures seven cognitive domains of particular relevance to SZ: speed of processing, attention/vigilance, working memory, verbal learning, visual learning, and reasoning, and problem-solving. The battery was designed for use as a repeated measure in clinical trials of pro-cognitive therapeutics. The MCCB scoring program yields individual domain scores (speed of processing, attention/vigilance, working memory, verbal learning, visual learning, and reasoning and problem solving) and a composite score. Clinical symptoms were assessed with the scale for the assessment of positive symptoms (SAPS)^50^ and negative symptoms (SANS)^51^. SAPS comprises individual symptom ratings that are divided among four subdomains (i.e., hallucinations, delusions, bizarre behavior, and formal thought disorder), each subdomain is also given a separate global symptom-severity score. SANS symptom ratings are divided among five subdomains (i.e., affective flattening/blunting, alogia, avolition–apathy, anhedonia–asociality, and attention), and are also given separate global symptom-severity ratings. Individual subdomains, and the composite and global scores for both SAPS and SANS, were calculated^52^. Motivation and pleasure (MAP) and expressive (EXP) dimensions of negative symptoms^53^ were quantified by averaging SANS subscales; MAP is the mean of Anhedonia and Apathy subscales, and EXP is the mean of Affective blunting and Alogia subscales.

### EEG paradigm, acquisition, and processing

The ASSR paradigm utilized 500-ms trains of 85-dB clicks (1-ms duration each) presented at a frequency of 40 Hz. A total number of 250 click trains were played with an intertrain interval of 0.5 s. The auditory stimuli were delivered through insert earphones. Participants were instructed to ignore auditory stimuli while watching a silent movie. ASSR recordings lasted approximately 4 min. EEG data were continuously recorded with a 64-channel BioSemi ActiveTwo system at a sampling rate of 8192 Hz. Data processing were performed offline using a precoded pipeline and applied to all subjects in an automated manner on BrainVision Analyzer as per established methods^17,43^. Briefly, data were downsampled to 1000 Hz. A robust average reference was applied to the EEG recordings, and eye-movement artifacts were corrected using independent component analysis. Continuous data were segmented relative to the onset of the stimuli (−500 to 500 ms), and each epoch was baseline-corrected relative to the 100-ms prestimulus interval. Epochs containing ±70 μV were automatically rejected. Gamma-evoked power (*γ*EP) and phase locking (*γ*PL) were averaged across frontocentral electrodes (C1, C2, Cz, F1, F2, Fz, FC1, FC2, and FCz) to create a composite frontocentral measure (FC Comp). *γ*EP and *γ*PL were calculated on wavelet coefficients obtained from the Morlet wavelet transformation of the segmented data (representing the 1–50-Hz frequency range, with a total number of 50 frequency layers using a Morlet parameter of 10). *γ*PL quantifies the consistency of the oscillatory phase across individual trials, ranging from 0 (purely non-phase-locked activity) to 1 (fully phase-locked activity). γPL was estimated by averaging across the 36–44-Hz frequency layers. Mean values obtained for each of the six 100-ms time windows from −100 to 500 ms relative to stimulus onset and the mean of the entire 500-ms post-stimulus interval were used in separate analyses described below.

### Statistical analyses

We first assessed whether 1 h of TCT or computer games had any temporal effects on ASSR activity (e.g., early vs. late) similar to what was seen in our previous studies^17,43^. Linear mixed-effect models were used to analyze the effects of 1 h of TCT on gamma oscillatory activity (*γ*EP and *γ*PL). Six 100-ms time windows from the –100- to 500-ms ASSR segment were used as dependent variables and were regressed onto contrast-coded treatment (TAU and TCT), time (T0 and T1), and interaction terms modeled as fixed effects. All models included centered fixed effects and subject intercepts as random effects. In contrast to our expectations, analysis with linear mixed-effect models did not reveal any significant *treatment × time* interactions on ASSR activity; hence, subsequent analyses were conducted using the mean response from the 1–500-msec post-stimulus window.

Analyses were focused on a priori-defined spatiotemporal ASSR measure (e.g., the 1–500-msec post-stimulus window derived from only the frontocentral composite electrode) and a change in key clinical and cognitive outcomes to reduce the number of statistical comparisons and the likelihood of type I error. We used linear regression models to assess the relationship between gamma oscillatory activity and TCT outcomes, where primary outcome variables were regressed onto ASSR measures of gamma oscillatory activity (e.g., baseline or “malleability” indices of *γ*EP and *γ*PL), treatment (TAU and TCT) group, and their interaction (e.g., ASSR predictor × treatment interaction). Primary outcome variables were: (1) a change in neurocognitive composite T scores (MCCB-NC), change in (2) SAPS, and (3) SANS composite measures. A change in primary outcome variables was calculated as a “T2 minus T0” difference score. “Malleability” of ASSR predictors was calculated as a “T1 minus T0” difference score over the 1–500-ms post-stimulus interval. Rank-based inverse normal transformations were applied to dependent variables when fitted-model residuals were non-normally distributed^54^. Only interaction terms were examined for significance and are reported as delta *R*^2^ (Δ*R*^2^). Delta *R*^2^ represents the improvement in *R*^2^ gained by the introduction of an interaction term in a regression model (i.e., the *R*^2^ for the model with the main effects of ASSR predictor, treatment, and their interaction *minus* the *R*^2^ for the model with the main effects of ASSR predictor and treatment alone). Significant ASSR × treatment interactions were decomposed via post hoc analysis of secondary outcome measures (e.g., subscales/subdomains of primary outcomes) and by follow-up regressions for each treatment group, separately. In other words, a significant ASSR predictor × treatment interaction on MCCB-NC would be followed up by (1) analyzing ASSR predictor × treatment interactions on MCCB subscales (e.g., attention/vigilance, working memory, etc.) and (2) by assessing the effect size (i.e., *R*^2^) of the ASSR predictor on primary and secondary outcome measures in the treatment groups, separately. Statistical analyses were implemented using the “*lme4*” package^55^ and built-in functions in R.

## Results

### Demographic and clinical features

The demographic and clinical characteristics of this sample were previously reported^33,46^ and are shown in Table 1. There were no significant differences in any demographic (e.g., age, age of onset, sex, education, and WRAT) or clinical variable (e.g., SANS, SAPS, chlorpromazine equivalents (CPZ), and global assessment of function (GAF)) between the treatment groups.

**Table 1:** Baseline demographic and clinical characteristics.

	TAU (*n* = 21) mean (sem)	TCT (*n* = 21) mean (sem)	*p*
Age (years)	33.2 (2.4)	35.6 (2.6)	*n.s*.
Sex (F:M)	11:10	11:10	*n.s*.
Age of onset (years)	21.0 (1.1)	18.6 (1.1)	*n.s*.
Education (years)	11.7 (0.5)	12.4 (0.4)	*n.s*.
WRAT	92.4 (3.0)	91.4 (3.0)	*n.s*.
*Scale for assessment of positive symptoms*			
Hallucinations	4.2 (1.5)	3.7 (1.1)	*n.s*.
Delusions	4.0 (1.3)	7.2 (1.8)	*n.s*.
Bizarre behavior	0.8 (0.3)	0.5 (0.2)	*n.s*.
Thought disorder	5.2 (1.5)	4.5 (1.4)	*n.s*.
Composite	14.2 (3.8)	15.8 (3.3)	*n.s*.
Global	4.8 (1.1)	5.0 (0.9)	*n.s*.
*Scale for assessment of negative symptoms*			
Affective blunting	7.4 (1.4)	8.4 (1.6)	*n.s*.
Alogia	2.5 (0.7)	2.4 (0.5)	*n.s*.
Apathy	2.0 (0.5)	2.0 (0.5)	*n.s*.
Anhedonia	3.7 (0.7)	3.4 (0.6)	*n.s*.
Attention	3.8 (0.7)	2.4 (0.5)	*n.s*.
Composite	19.3 (2.7)	18.7 (2.7)	*n.s*.
Global	6.9 (0.9)	7.5 (1.0)	*n.s*.
Motivation and pleasure	2.8 (0.4)	2.7 (0.5)	*n.s*.
Expressive symptoms	5.0 (0.9)	5.4 (0.9)	*n.s*.
GAF	30.5 (1.1)	31.1 (1.5)	*n.s*.
Chlorpromazine equivalents	946.7 (176.8)	1154.8 (245.2)	*n.s*.

Demographics and clinical symptoms.

### ASSR biomarkers do not significantly change after 1 h of TCT or computer games

Linear mixed-effect analysis did not find any significant treatment × time interactions on *γ*EP (*β* = 0.01, SE = 0.03, *t* = 0.47, *p* > 0.5) or *γ*PL (*β* = –0.02, SE = 0.04, *t* = –0.69, and *p* = 0.50) at the group level.

### Baseline γEP predicts cognitive improvement

**Fig. 1:**
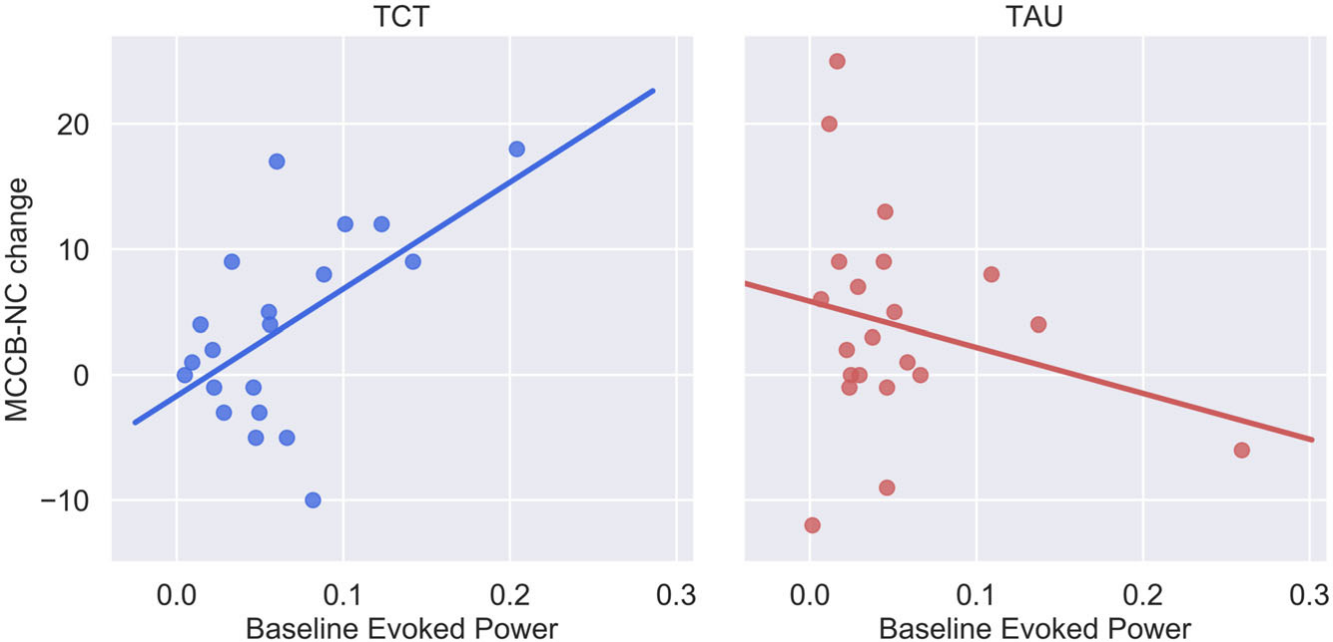
Baseline-evoked gamma power predicts overall TCT-related cognitive improvement. The significant interaction between baseline-evoked power and treatment on change in MCCB-NC (Δ*R*^2^ = 0.16, *p* = 0.01), revealed that the effect was driven by the TCT group (*R*^2^ = 0.31), but not the TAU group (*R*^2^ = 0.06).

Linear regression analysis revealed a significant interaction between baseline *γ*EP and improvement in overall neurocognitive functioning in the TCT group relative to the TAU group (MCCB-NC; Δ*R*^2^ = 0.16, *β* = 0.40, SE = 0.15, and *p* = 0.012, Fig. 1). Secondary analyses on MCCB subscales revealed that this omnibus cognition effect was likely driven by significant interactions between baseline *γ*EP and treatment group for the attention and vigilance (Δ*R*^2^ = 0.18, *β* = 0.38, SE = 0.15, and *p* = 0.015) and working memory (Δ*R*^2^ = 0.17, *β* = 0.31, SE = 0.15, and *p* = 0.050) subdomains. No significant interactions were found between baseline *γ*EP and treatment on either clinical symptom outcomes or baseline *γ*PL and treatment on any primary cognitive or clinical outcome variables. Table 2 summarizes the results of ASSR × treatment interactions and follow-up analyses of primary outcomes. Supplementary Table 1 summarizes the results of ASSR × treatment interactions and follow-up analyses of secondary outcomes.

**Table 2:** Relationships between gamma oscillatory biomarkers and TCT-related change in cognitive and clinical outcomes.

	TCT *R*^2^	TAU *R*^2^	ASSR × treatment interaction				
			Δ*R*^2^	*β*	SE	*t*	*p*
*Baseline gamma power*							
Δ MCCB-NC	0.31	0.06	0.16	0.40	0.15	2.64	0.012
Δ SANS composite	0.03	0.01	0.02	–0.15	0.17	–0.87	0.390
Δ SAPS composite	0.16	0.00	0.00	–0.06	0.16	–0.39	0.699
*Baseline gamma phase-locking*							
Δ MCCB-NC	0.15	0.05	0.08	0.30	0.16	1.83	0.075
Δ SANS composite	0.02	0.01	0.01	–0.13	0.18	–0.73	0.473
Δ SAPS composite	0.09	0.02	0.00	–0.06	0.16	–0.38	0.703
*1-h Δ Gamma power*							
Δ MCCB-NC	0.19	0.01	0.07	–0.26	0.16	–1.62	0.115
Δ SANS composite	0.30	0.04	0.15	–0.39	0.15	–2.53	0.016
Δ SAPS composite	0.40	0.04	0.14	0.38	0.15	2.59	0.014
*1-h Δ Gamma phase locking*							
Δ MCCB-NC	0.00	0.06	0.02	0.14	0.17	0.86	0.396
Δ SANS composite	0.20	0.15	0.00	–0.03	0.16	–0.16	0.871
Δ SAPS composite	0.20	0.24	0.24	0.50	0.14	3.52	0.001

The primary analysis focused on elucidating significant ASSR biomarker × treatment interactions on TCT-related change in cognitive and clinical outcomes using linear regressions. To further clarify any significant interactions, *R*^2^ values are also provided for linear regressions run in TCT and TAU groups, separately.

### “Malleability” indices predict improvement in clinical symptoms

**Fig. 2:**
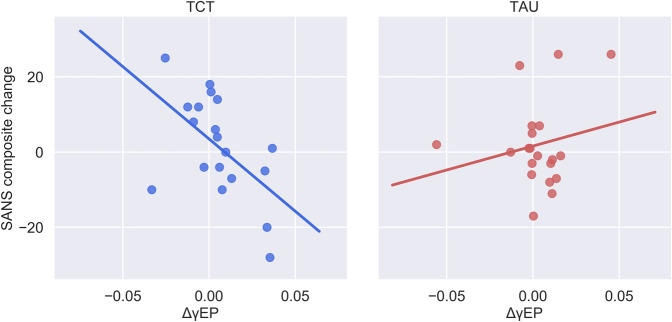
Malleability of gamma-evoked power after 1 h of TCT predicts overall improvement in negative symptoms. Decomposing the significant ΔγEP × treatment interaction (Δ*R*^2^ = 0.15, *p* = 0.01) on change in SANS symptoms revealed that the effect was largely driven by the TCT group (*R*^2^ = 0.30), but not the TAU group (*R*^2^ = 0.04).

**Fig. 3:**
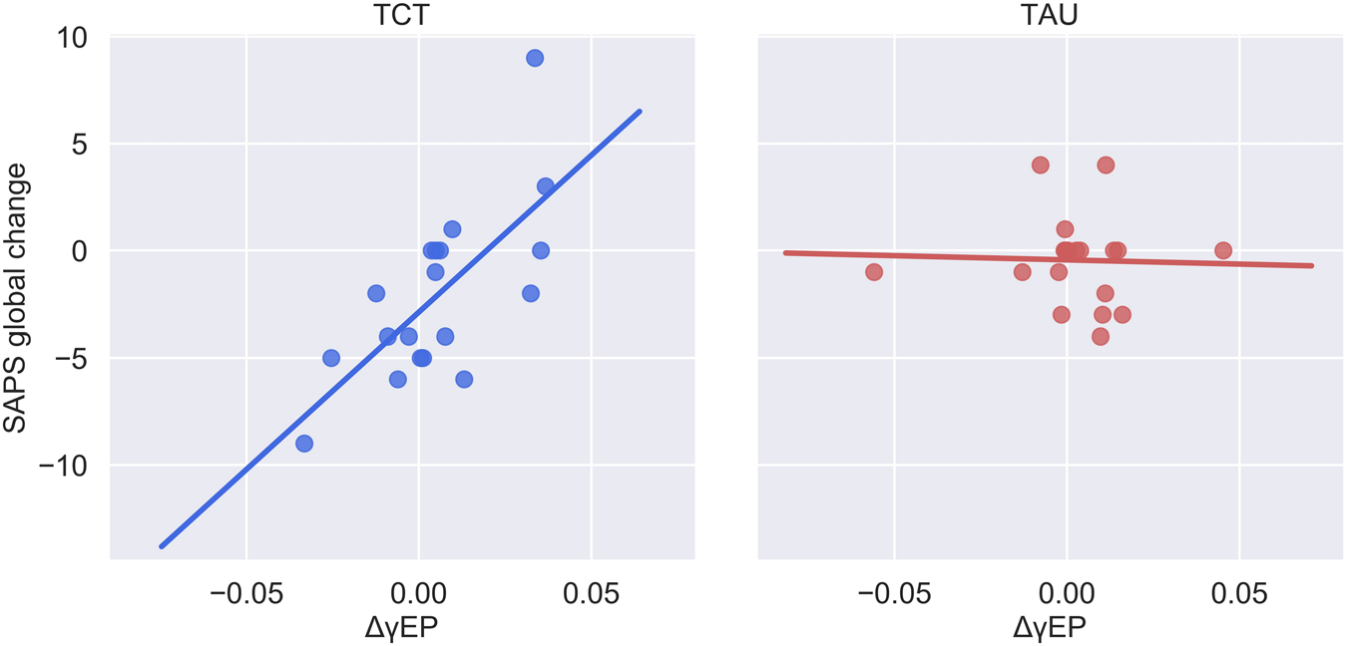
Malleability of gamma-evoked power after 1 h of TCT predicts overall improvement in global positive symptom severity. Decomposing the significant ΔγEP × treatment interaction on change in SAPS Global (Δ*R*^2^ = 0.10, *p* = 0.002) revealed that the effect was largely driven by the TCT group (*R*^2^ = 0.49) and was absent in the TAU group (*R*^2^ < 0.01).

The magnitude of change in gamma power after 1 h of TCT (ΔγEP) significantly predicted clinical improvement in overall positive (SAPS composite: Δ*R*^2^ = 0.14, *β* = 0.38, SE = 0.15, and *p* = 0.014) and negative (SANS composite: Δ*R*^2^ = 0.19, *β* = –0.38, SE = 0.15, and *p* = 0.016, Fig. 2) symptoms at the end of the trial in the TCT group relative to TAU. To follow up on the primary findings on overall psychopathology, secondary analyses of individual SAPS and SANS subscales were conducted. This omnibus effect of ΔγEP and SAPS was likely driven by changes in Hallucinations (Δ*R*^2^ = 0.14, *β* = 0.36, SE = 0.14, and *p* = 0.017) and global symptom severity (Δ*R*^2^ = 0.19, *β* = 0.44, SE = 0.13, and *p* = 0.002, Fig. 3). ΔγEP also predicted a change in individual SANS subdomains, including anhedonia (Δ*R*^2^ = 0.10, *β* = –0.32, SE = 0.16, and *p* = 0.05) and EXP-negative symptoms (i.e., the mean of affective blunting and alogia subscales^53^) (Δ*R*^2^ = 0.11, *β* = –0.34, SE = 0.16, and *p* = 0.038). ΔγEP did not interact significantly with any primary cognitive outcomes.

The magnitude of change in gamma phase-locking after 1 h of TCT (ΔγPL) significantly predicted clinical improvement in overall positive symptoms (SAPS composite: Δ*R*^2^ = 0.24, *β* = 0.50, SE = 0.14, and *p* = 0.001) in the TCT group relative to TAU. ΔγPL was also found to predict changes in SAPS subscales, including a change in Hallucinations (Δ*R*^2^ = 0.10, *β* = 0.29, SE = 0.14, and *p* = 0.048), Delusions (Δ*R*^2^ =0.11, *β* = 0.34, SE = 0.14, and *p* = 0.019), Thought Disorder (Δ*R*^2^ = 0.15, *β* = 0.40, SE = 0.16, and *p* = 0.017), and Global Symptom Severity (Δ*R*^2^ = 0.15, *β* = 0.39, SE = 0.14, and *p* = 0.010). ΔγPL did not interact significantly with primary cognitive or negative-symptom outcome measures.

## Discussion

Developing effective treatments for the hallmark cognitive symptoms of SZ continues to be one of the more daunting challenges in psychiatric neuroscience. While antipsychotic medications provide modest relief of the more dramatic, but “secondary” symptoms (e.g., hallucinations and delusions) of SZ^56^, the core cognitive symptoms persist and cause profound disability and loss of functioning for many patients^30,57,58^. Auditory-based TCT is a promising approach to cognitive remediation, although considerable interindividual variability in treatment response has limited the translation of TCT from academic laboratories to real-world community settings. The present study assessed whether ASSR measures of gamma power and synchrony collected at the outset of treatment would predict the cognitive and clinical benefits of TCT in a cohort of SZ patients receiving long-term care in a community-based residential treatment facility. Specifically, the findings suggest that baseline *γ*EP is a significant predictor of TCT-induced cognitive enhancement and that the “malleability” or plasticity of gamma power and synchronization immediately following acute exposure to the initial 1-h “dose” of TCT robustly predicts changes in psychopathological dimensions.

Currently, there is a dearth of reliable biomarkers that can predict future cognitive training-related gains either at baseline or early in the treatment course^47,59,60^. Hence, a significant proportion of patients who are treated with TCT do not demonstrate any meaningful improvement in cognitive functioning or clinical symptoms. The relationship between baseline γEP and improvement in global cognition after 30 h of TCT suggests that a brief (i.e., 4–5 min) ASSR assessment can predict the extent of pro-cognitive gains from a full (30 h) course of TCT. While these data are insufficient for providing a mechanistic understanding of the changes in brain function attributable to TCT, this particular form of cognitive remediation is designed to leverage intact neuroplasticity mechanisms in “spared” neural circuitry rather than promoting compensatory strategies for working around their cognitive impairments. Baseline gamma-evoked power may be an index of the brain’s overall “adaptive integrity“ in these lower-level perceptual networks as the patients with the largest ASSR responses had more robust cognitive gains in response to TCT^39,44,61^.

Previous studies support the notion that auditory-based TCT mediates its therapeutic effects on neurocognition by inducing neuroplastic changes in distributed frontal–temporal and thalamocortical circuits^25,62–65^. Indeed, training-induced changes in oscillatory activity and event-related potentials have been shown to correlate with TCT-induced cognitive enhancements^62,65^. These training-related effects on gamma activity (i.e., the pre–post difference scores after 30–50 hours of TCT) have been shown to correlate with improvements in executive function and other cognitive domains^62,65^. Similar patterns of adaptive neuroplasticity have been reported for EAIP biomarkers in response to as little as 1 h of TCT and acute pharmacologic challenges^41,42,66^. Previous studies using mismatch negativity (MMN) provide empirical support for the utility of EAIP biomarkers in the prediction of TCT-related cognitive outcomes^46,47^. While baseline MMN measures alone do not appear to be particularly sensitive in detecting the pro-cognitive effects of TCT in this cohort of patients^47,60,67^, the “malleability” of MMN responses after 1 h of TCT were sensitive to the acute learning effects of TCT and response to a full (30-h) course of TCT^46^. It is conceivable that the neuroplasticity mechanisms indexed by ASSR and other EAIP biomarkers may be used synergistically to predict future benefits in the overall neurocognitive and clinical status and/or gains in specific neurocognitive and clinical domains.

Interestingly, though 1 h of TCT did not produce any statistically significant changes in evoked gamma power and synchrony at the group level in frontocentral electrodes, we found that changes after 1 h of training at the individual level explained significant amounts of variance in the change in symptom domains (see Table 2 and Supplemental Table 1). Specifically, ΔγEP explained ~30–50% of the variance of the change in multiple SAPS subdomains after a full course of TCT. Measures of ΔγPL also predicted a change in SAPS and subdomains of positive symptoms, but not as robustly as ΔγEP. Similarly, the malleability of evoked gamma power predicted an overall change in negative- symptom severity. Two independent meta-analyses of cognitive remediation trials have reported small effect sizes (*g* ≈0.1–0.3) on negative symptoms^36,38^, suggesting that subsets of patients may experience modest clinical improvement in this symptom domain. While TCT did not produce a significant improvement in negative symptoms at the group level in this cohort of patients^33^, these findings suggest that ASSR biomarkers may predict beneficial treatment response in terms of negative symptoms in subsets of patients who undergo TCT. These results extend our previous findings of EAIP malleability as predictors of TCT-related change in positive-symptom severity in this same cohort of patients^46^ to include ASSR-evoked gamma oscillatory biomarkers as robust predictors of clinical outcomes in response to TCT. These patterns of ASSR malleability predicting improvement in psychopathological dimensions are complex and should be interpreted cautiously. Further studies incorporating ASSR biomarkers in experimental medicine trials of TCT and other pro-cognitive interventions are necessary to replicate and extend the present findings.

### Caveats and limitations

Findings from the current study should be considered in the context of several important limitations. First, the sample sizes of this “proof-of-concept” cognitive remediation trial were modest. While this sample was sufficiently powered to detect moderate-to-large effect sizes^33^, the overall sample size was not powered to identify other mediators or moderators of therapeutic gains, or to tease apart complex multivariate relationships in this data set. Although the large effect sizes of the findings in the TCT group warrant further investigation and replication, it is curious that ASSR biomarkers were not robust predictors of target engagement for computer games or general outcomes associated with TAU. This pattern of findings supports the interpretation that ASSR is a sensitive and specific biomarker of TCT response. Second, medication status was not experimentally controlled; patients were receiving complex medication regimens per “standard of care” community-based practices for treatment-refractory psychosis. While we cannot rule out the potential for medications to enhance or blunt gains from cognitive training, our previous studies from this sample suggest that the pro-cognitive benefits of TCT may offset the adverse cognitive effects of antipsychotic polypharmacy and anticholinergic burden^68^. Third, this study was conducted in patients with refractory psychotic disorders with well-established illness; therefore, the findings may not generalize to at-risk, early illness, or other clinical populations^28,69^. Fourth, no group-level differences were detected in ASSR measures after 1 h of TCT. While these results were unexpected, it is possible that the “malleability” of ASSR responses may not be detectable at the level of scalp electrodes. Scalp-level ASSR responses are generated by dynamic interactions from a distributed network of frontotemporal sources;^25^ it is conceivable that 1 h of TCT may induce plasticity in discrete sources and/or their dynamic connectivity patterns, which may not be readily apparent when collapsing across the full 1–500-ms response window at a frontocentral composite electrode. Fifth, even with the emphasis on distilled composite measures, relationships among ASSR measures and TCT-related outcomes are still complex. Future larger-scale clinical trials are needed to better characterize the neural substrates of ASSR biomarkers and the relationships with TCT-related pro-cognitive and clinical outcomes.

In this context, another limitation of relevance to clinical and translational studies incorporating high-density EEG (or other high-dimensional brain phenotypes) as predictive biomarkers of treatment outcomes in clinical trials is the need to rely upon relatively circumspect and conservative (e.g., frequentist) analytic approaches. Analyses in this proof-of-concept study were constrained to a few a priori predictors and outcomes in order to minimize the risk of type 1 errors. Despite this conservative statistical framework, multiple “signals” and potentially clinically relevant associations were detected, most of which were specific to the TCT group. It is possible (and likely) that while attempting to mitigate the risk of type 1 error, many associations between ASSR variables (e.g., at discrete time windows or other electrode sites) and clinically and cognitively relevant outcomes were missed (i.e., type 2 error). As high-dimensional neurobiological measures are increasingly applied for use as predictive biomarkers of therapeutic outcomes in clinical trials, novel statistical methods that allow for the detection of meaningful associations embedded within multidimensional clinical datasets that emphasize effect sizes and appropriately balance the risk of type 1 and type 2 errors are necessary. These methodological developments will be critical to the advancement of biologically informed treatments for SZ and other neuropsychiatric disorders.

## Conclusions

To our knowledge, this is the first study to demonstrate the feasibility of ASSR biomarkers as clinically relevant predictors of cognitive and clinical outcomes in community settings. These findings underscore the utility of incorporating neurophysiologic biomarkers of gamma-evoked power and synchronization in the development of pro-cognitive therapeutics and their potential application in routine clinical practice. It is conceivable that the coupling of validated behavioral^40,70–72^ and neurophysiologic measures of target engagement^46,47,59,60^ may enhance the sensitivity and accuracy of predictive algorithms for TCT-related cognitive and clinical outcomes. These findings thus support future personalized medicine trials of pro-cognitive therapeutics, where individuals may be stratified to receive effective interventions based on their unique neurophysiologic profiles and their likelihood of response.

## Supplementary information

Supp Table 1
