# Supplementary material for: Gamma oscillations predict pro-cognitive and clinical response to auditory-based cognitive training in schizophrenia
Source: Transl Psychiatry. 2020 Nov 23;10:405. doi: 10.1038/s41398-020-01089-6 (PMC7684295; doi:10.1038/s41398-020-01089-6)
Supplement: Supplementary file 1 — Supp Table 1 [file 41398_2020_1089_MOESM1_ESM.docx]

**Supplemental Table 1.** Summary of secondary outcome measures. The analytic strategy first required a significant ASSR x treatment interactions on primary outcomes. These interactions were further decomposed by assessing interaction effects on secondary outcomes (or subscales of the primary outcome measure) and follow up regressions separately in each group.

|  |  | **TCT** | **TAU** | **ASSR x Treatment Interaction** | | | | |
| --- | --- | --- | --- | --- | --- | --- | --- | --- |
| **Predictor** | **Outcome** | *R^2^* | *R^2^* | *ΔR2* | *β* | *SE* | *t* | *p* |
| **Baseline Gamma Power** |  |  |  |  |  |  |  |  |
| Baseline Gamma Power | Δ MCCB-NC | 0.31 | 0.06 | 0.16 | 0.40 | 0.15 | 2.64 | 0.012 |
| Baseline Gamma Power | Δ MCCB-Speed of Processing | 0.26 | 0.00 | 0.06 | 0.24 | 0.16 | 1.51 | 0.140 |
| Baseline Gamma Power | Δ MCCB-Attention/Vigilance | 0.27 | 0.03 | 0.14 | 0.38 | 0.15 | 2.54 | 0.015 |
| Baseline Gamma Power | Δ MCCB-Working Memory | 0.03 | 0.20 | 0.09 | 0.31 | 0.15 | 2.03 | 0.050 |
| Baseline Gamma Power | Δ MCCB-Verbal Learning | 0.03 | 0.00 | 0.01 | 0.07 | 0.17 | 0.45 | 0.655 |
| Baseline Gamma Power | Δ MCCB-Visual Learning | 0.06 | 0.02 | 0.01 | 0.11 | 0.16 | 0.69 | 0.494 |
| Baseline Gamma Power | Δ MCCB-Reasoning/Prob. Solving | 0.04 | 0.16 | 0.08 | 0.29 | 0.16 | 1.80 | 0.079 |
|  |  |  |  |  |  |  |  |  |
| **1-h Δ Gamma Power** |  |  |  |  |  |  |  |  |
| 1-h Δ Gamma Power | Δ SAPS Composite | 0.35 | 0.02 | 0.14 | 0.38 | 0.15 | 2.59 | 0.014 |
| 1-h Δ Gamma Power | Δ SAPS Hallucinations | 0.36 | 0.02 | 0.14 | 0.36 | 0.14 | 2.50 | 0.017 |
| 1-h Δ Gamma Power | Δ SAPS Delusions | 0.12 | 0.00 | 0.01 | 0.11 | 0.15 | 0.74 | 0.464 |
| 1-h Δ Gamma Power | Δ SAPS Bizarre Behavior | 0.00 | 0.12 | 0.04 | 0.19 | 0.15 | 1.32 | 0.197 |
| 1-h Δ Gamma Power | Δ SAPS Thought Disorder | 0.15 | 0.02 | 0.06 | 0.25 | 0.16 | 1.56 | 0.127 |
| 1-h Δ Gamma Power | Δ SAPS Global | 0.49 | 0.00 | 0.19 | 0.44 | 0.13 | 3.39 | 0.002 |
|  |  |  |  |  |  |  |  |  |
| 1-h Δ Gamma Power | Δ SANS Composite | 0.30 | 0.04 | 0.15 | -0.39 | 0.15 | -2.53 | 0.016 |
| 1-h Δ Gamma Power | Δ SANS Affective Blunting | 0.22 | 0.01 | 0.09 | -0.30 | 0.16 | -1.94 | 0.061 |
| 1-h Δ Gamma Power | Δ SANS Alogia | 0.10 | 0.04 | 0.06 | -0.26 | 0.17 | -1.56 | 0.128 |
| 1-h Δ Gamma Power | Δ SANS Apathy | 0.10 | 0.00 | 0.02 | -0.15 | 0.16 | -0.90 | 0.375 |
| 1-h Δ Gamma Power | Δ SANS Anhedonia | 0.04 | 0.20 | 0.10 | -0.32 | 0.16 | -2.03 | 0.050 |
| 1-h Δ Gamma Power | Δ SANS Attention | 0.03 | 0.07 | 0.01 | 0.09 | 0.15 | 0.56 | 0.578 |
| 1-h Δ Gamma Power | Δ SANS Global | 0.05 | 0.04 | 0.04 | -0.20 | 0.16 | -1.24 | 0.222 |
| 1-h Δ Gamma Power | Δ SANS MAP | 0.07 | 0.15 | 0.10 | -0.31 | 0.15 | -2.01 | 0.052 |
| 1-h Δ Gamma Power | Δ SANS EXP | 0.24 | 0.02 | 0.11 | -0.34 | 0.16 | -2.16 | 0.038 |
|  |  |  |  |  |  |  |  |  |
| **1-h Δ Gamma Phase Locking** |  |  |  |  |  |  |  |  |
| 1-h Δ Gamma Phase Locking | Δ SAPS Composite | 0.24 | 0.23 | 0.24 | 0.50 | 0.14 | 3.52 | 0.001 |
| 1-h Δ Gamma Phase Locking | Δ SAPS Hallucinations | 0.06 | 0.17 | 0.10 | 0.29 | 0.14 | 2.05 | 0.048 |
| 1-h Δ Gamma Phase Locking | Δ SAPS Delusions | 0.07 | 0.24 | 0.11 | 0.34 | 0.14 | 2.46 | 0.019 |
| 1-h Δ Gamma Phase Locking | Δ SAPS Bizarre Behavior | 0.03 | 0.00 | 0.00 | 0.05 | 0.15 | 0.30 | 0.763 |
| 1-h Δ Gamma Phase Locking | Δ SAPS Thought Disorder | 0.14 | 0.17 | 0.15 | 0.40 | 0.16 | 2.52 | 0.017 |
| 1-h Δ Gamma Phase Locking | Δ SAPS Global | 0.17 | 0.23 | 0.15 | 0.39 | 0.14 | 2.73 | 0.010 |
